# Heterogeneous Returns to Social Networks: Effects on Earnings and Job Satisfaction in the Chinese Labor Market

**DOI:** 10.3390/ijerph19095700

**Published:** 2022-05-07

**Authors:** Xiaoxian Guo, Qi Chen

**Affiliations:** 1 Department of Sociology, Institute for Empirical Social Science Research, School of Humanities and Social Science, Xi’an Jiaotong University, Xi’an 710049, China; 2 Qu Qiubai School of Government, Changzhou University, Changzhou 213164, China; raymondcq@cczu.edu.cn

**Keywords:** social networks, heterogeneous treatment effects model, heterogeneous returns, earnings effect, job satisfaction effect

## Abstract

With the advancement of social network research over time, a research consensus has been reached that the use of social networks in job searching can provide positive returns. This study focused on the heterogeneity in the returns to social networks. Using Job Search and Social Networks (JSNET) survey data on urban residents of China, we examined the differences in the propensity to use social networks in job searching and the returns to social networks in terms of job seekers’ earnings and job satisfaction using propensity score stratification and the heterogeneous treatment effects model (HTE model). The use of social networks in job searching was found to be nonrandomly distributed, and the propensity to use such networks varied according to job seeker characteristics. For job seekers with different propensities, the returns to social networks were also different. Moreover, there was negative selection in the instrumental effect of social network use in the job search process but positive selection in the expressive effect: the higher the propensity to use one’s networks, the lower the income return to social networks and the higher the job satisfaction; the lower the propensity to use one’s networks, the higher the income return and the lower the job satisfaction.

## 1. Introduction

Numerous studies have demonstrated that social networks have a positive effect on finding a job [1,2,3,4]. Along with the gradual introduction of social network analysis into research on China, many studies have directly focused on the Chinese labor market [5,6,7,8,9,10,11,12,13]. In general, job seekers who use social networks are able to attain higher earnings and a better occupational status than job seekers who do not use social networks. Studies on China also tend to place more emphasis on the institutional environment, linking social network analysis to the market reform process [7,11]. As an informal source of resource allocation, social networks play an important role alongside formal sources such as the market and the government. In the early stages of China’s market reform, when the rules and norms of the market were not sufficiently clear, the role of social networks as informal channels for resource allocation gradually increased. As marketization deepened over time, the market rules became clearer and stronger, and the space for network-based activity declined. This means that the effect of social networks followed an inverse U-shaped trend (first increasing and then decreasing) as marketization intensified over time.

However, recent research has shown that the percentage of individuals who use social networks when searching for a job does not follow an inverse U-shaped pattern as supposed; rather, it has continued increasing and, according to recent data analysis, has reached 80% (JSNET data shows that 78.7% of job seekers have used social networks when searching for a job during China’s marketization period, which began in 2002). This means that it is now common to obtain help from one’s social networks when seeking a job. We have strong reason to believe that those actors who use their social networks when seeking a job are not a small, special group; given that nearly 80% of job seekers utilize their social networks, the heterogeneity within this group is likely very large. Against the background of an increasing percentage of job seekers using social networks and the increase in heterogeneity, a new but very important question arises: Are the returns to social networks among those actors who use social networks when seeking a job consistent? When we focus on job seekers who use social networks, do the returns to social networks vary from person to person or group to group? This question discusses the return of social networks, which is in fact the endogeneity of social networks. Endogeneity has been a hot topic in the fields of social networks and social capital, and the existence of endogeneity has led the scholars to doubt whether there are really returns to social capital accordingly. In this study, we fully examine the complete causality and further point out the existence of group differences in the returns to social capital while discussing the returns to social capital. This will help us to answer the endogenous challenge of social networks and help us to have a more complete and accurate understanding of the returns of social networks in the labor market.

## 2. Effects of Social Networks: The Origin of Heterogenous Returns

Social network analysis has attracted abundant interest in and research on the effects of social networks since the 1970s, the beginning of the rise of social network research. Regarding whether social networks play a role in the labor market and, if so, how, various theoretical perspectives, such as the network strength approach [1,4], network resource approach [3], network structure approach [14], and network signal approach [15], have been discussed. Whether considering strong or weak ties under the network strength approach, information or favorable treatment under the network resource approach, or structural holes under the network structure approach, the effect of social networks has been comprehensively demonstrated. Among the many fields that have incorporated social network analysis, the study of the labor market has always been at the forefront of social network analysis, and the job-search process is a good context in which to examine the role of social networks. As discussed above, the effects of social networks may be diverse, as most job seekers use social networks [16]. When exploring the role of social networks, a failure to account for differences in the relationship between social networks and status attainment imposes limitations. When we move away from an overall analytical perspective and instead analyze the differences in the returns to social networks, then there are two possible theoretical assumptions: positive returns and negative returns.

The social capital embedded in social networks has both instrumental and expressive effects [17]. Most of the previous studies on the effects of social networks in the labor market have focused on the income effects of social networks. In fact, the process of acquiring status in the labor market and the corresponding outcomes involve both the instrumental effects represented by income and the expressive effects of the job search process and its outcome. One of the most direct expressive effects is job satisfaction after the job search. Job satisfaction refers to the degree of satisfaction with the job found and is a subjective evaluation made by the job seeker. It is a global evaluation and may include dimensions such as satisfaction with the job itself, satisfaction with the salary package, satisfaction with the opportunities for promotion, and satisfaction with colleague relationships. The economic behavior of actors is embedded within a social structure, and job seekers might not simply choose the job with the highest salary and income when seeking an occupation but may be motivated by a global evaluation of various other non-instrumental indicators, such as colleague relationships and career development. Therefore, when we analyze the returns to social networks, we should not limit ourselves to the instrumental indicator of the returns in terms of earnings but should consider both instrumental and expressive returns. Both of these dimensions have two possible theoretical relationships with the use of social networks: positive return or negative return.

### 2.1. Positive Returns Hypothesis

In the social capital theory, Nan Lin describes a pyramidal social structure in which each actor is located at a certain point on the pyramid [3]. Social resources also exhibit this pyramidal structure. The higher up the actors are within this structure, the fewer positions and occupiers there are [3]. There are very few positions and occupiers at the top level, where those who control the largest number of valuable resources are located. The further up the structure one goes, the fewer actors and the more resources there are. At the bottom of the structure, the number of actors is larger, and the resources are fewer. In his theory, Lin proposed the “strength-of-position” proposition: the better the position of origin is, the more likely the actor is to access and use social capital, which is likely to be of higher quality [17].

The strength-of-position proposition is consistent with traditional structural theory, which describes the structural advantages of each actor and extends to social capital research. The initial position refers to both the actor’s ascribed position and achieved position. The ascribed position is the position inherited by the individual from their parents and relatives; the achieved position refers to the social position acquired and occupied through self-effort. A better initial position results not only in more social resources being available to the actor but also in human capital, political capital, and family background advantages, among others. When social capital is combined with other advantages, it can result in combined advantages such that the whole is greater than the sum of its parts.

However, actors with an average or worse initial position are at a double disadvantage: on the one hand, due to structural constraints, the social resources that they can mobilize through their social networks are inferior in both quantity and quality to those of actors with a better initial position. On the other hand, they also lack other relevant advantages that could be combined with their social capital in order to exploit the combined advantages.

When an actor has a weak endowment of all types of capital, the whole may be less than the sum of its parts. In summary, the better the initial position of the actor, the more likely that actor is to use their social network. Moreover, the more likely the actor is to use their network, the greater the return from the social network is likely to be. In this paper, we take current employment earnings and job satisfaction as the returns of getting a job. So, we state the research hypothesis of positive returns to social networks as follows:

**Hypothesis** **1.1:** *The more likely an actor is to use their social networks, the higher the current employment earnings they would obtain.*

**Hypothesis** **1.2:** *The more likely an actor is to use their social networks, the more satisfied with work they would be.*

### 2.2. Negative Returns Hypothesis

Bourdieu proposed the concept of social capital in contrast to economic capital and cultural capital at the beginning of his first elaboration of capital in “The Forms of Capital” [18]. Social capital, similar to economic capital, human capital, and cultural capital, is one of “the forms of capital”, and the return to such capital in terms of the income of job seekers may exhibit diminishing marginal effects with an increase in capital types. The importance of social capital is relatively minor when the actor has a sufficient number of other types of capital that can generate returns, while the importance of social capital is inevitably higher when the actor has access to fewer types of capital or even to social capital only.

Returning to the pyramidal social structure described by Nan Lin, those actors who are initially in a better position have relatively superior social capital, human capital, and political capital. According to the previous literature, human capital and political capital have a positive effect on actors in the Chinese labor market [19,20,21]. Therefore, in addition to social capital, other types of capital, such as human capital and political capital, provide higher rewards to those actors who are initially better positioned. The return to the social capital embedded in social networks is a return to only one of many types of capital and is simply icing on the cake.

Accordingly, for those actors with a poor initial position, the returns to social capital account for a greater share of their overall earnings due to their relative lack of other types of capital. The use or nonuse of social networks has a relatively greater or even decisive impact on such actors’ earnings. For these actors, the returns to social capital obtained through the use of social networks are relatively more important and can often be a much-needed helping hand. Therefore, the better the initial position of the actor is, the more likely that actor is to use his or her social network. However, the less likely the actor is to use his or her network, the greater the returns to the social network are likely to be. Accordingly, we obtain our research hypothesis on the negative returns to social networks.

**Hypothesis** **2.1:** *The less likely an actor is to use their social networks, the higher the current employment earnings they would obtain.*

**Hypothesis** **2.2:** *The less likely an actor is to use their social networks, the more satisfied with work they would be.*

### 2.3. Two Types of Selection Bias: Who Prefers to Use Social Networks? Who Benefits More from Social Networks?

#### Pretreatment and Treatment Effect Heterogeneity

On the basis of the principles of homogeneity and endogeneity, Ted Mouw raised suspicion of the social network effect in 2003 [22,23]. This resulted in a great deal of social network research in response to Mouw’s challenge [24]. A variety of methods have been used to address endogeneity. These include the Heckman two-step selection model, propensity score matching, instrumental variables, simultaneous equations system, and suspicion [25,26,27,28,29,30,31]. Recently, Brand, Yu, and their colleagues pointed out in a series of studies that when we want to accurately estimate a causal relationship, we should consider all of the endogeneity along the causal chain: the pretreatment heterogeneity bias and the treatment-effect heterogeneity bias [32,33,34]. Specifically, first, people are not randomly assigned to an experimental group (W = 1) and a control group (W = 0); rather, people with certain characteristics are more likely to become members of the experimental group. On the other hand, people with these characteristics are not only more likely to be in the experimental group, but they are also likely to obtain benefits that differ from those of their peers without these characteristics [33,35].

Endogeneity is a universal and unavoidable problem in all social sciences. However, since Mouw’s criticism of how endogeneity has been handled in social network analysis, the approach has been questioned and challenged. This is because the establishment of social networks—interactions among actors—is always purposeful and highly subjective. This makes it difficult for samples to satisfy the requirement that treatment status be randomly distributed and leads to estimation bias in the regression analysis. Given this difficulty, combined with the challenge of the complete endogeneity along the causal chain mentioned above, both of which affect the research on social networks in job search, we need to rethink the returns to social networks in the labor market. If there is endogeneity bias in the form of pretreatment heterogeneity bias, then people with certain characteristics are more likely to use social networks in when job searching. If there is endogeneity bias in the treatment effects, then people with those certain characteristics are not only more likely to use social networks but the returns that they obtain from their social networks are likely to vary, either increasing or decreasing with an increase in the likelihood of use. These two types of endogeneity bias are exactly what we try to analyze in this paper. Recent research from China has pointed out that the use of social networks in job searching is not randomly distributed and that those people who use social networks share common behavioral traits [36]. Actors who are strongly relational are more likely to use social networks when searching for a job. A preliminary study focused on investment in everyday relationships to discuss relational tendencies [36]. It has been demonstrated that the more actors invested in everyday relationships, the higher their tendency to use *guanxi* (social networks) when engaging in instrumental actions, such as searching for a job. Apart from attitudes toward relationalism, are there any other external characteristics that identify a behavioral tendency toward relationalism?

However, there is still a further problem: since social networks are not randomly selected, are the returns to social networks the same among different groups of people? This paper focuses on those people who use social networks in their job search and explores whether the effects of social networks vary from person to person. Specifically, in light of the two competing theoretical hypotheses proposed above, is it true that the more likely people are to use social networks, the higher the returns to social networks are? Or is it the case that the more likely people are to use social networks, the lower the returns are?

## 3. Data, Measures, and Methods

### 3.1. Data

We used data from the 2014 Job-Search Networks (JSNET2014) survey to examine the heterogeneity in the effects of social networks on job search. JSNET2014 contains data on individuals residing in 8 large cities (Changchun, Tianjin, Jinan, Xiamen, Shanghai, Guangzhou, Lanzhou, and Xi’an) throughout China that is representative data with a total sample size of 5480. This survey used probability proportional to size (PPS) sampling, in which a random sample was taken from three levels (district, subdistrict, and neighborhood) for each city. In each randomly selected neighborhood, households were selected from a household list through a systematic sampling technique. To obtain a complete list of both permanent and migrant households, a map of each neighborhood was drawn, and a list of the households that live there was made. From each randomly sampled household, an adult aged 18–69 was selected to be the respondent by randomly selecting birthdays. Above all, JSNET provides a representative sample and reliable results.

The JSNET data are an excellent source of information about social networks and job search, which are the key variables in our research. Unfortunately, there are no more data on job search networks update after 2014. Therefore, so far, these data are the most appropriate and the latest data on the measurement of social networks and occupationally related variables.

### 3.2. Measures

#### 3.2.1. Dependent Variable

**Earnings.** “Earnings” here refers to current employment earnings, which are the occupational earnings of the worker at the beginning of their entry into their current job. This study focuses on the relationship between the social networks used by job seekers in their job search and the subsequent occupational earnings obtained, and to strictly preserve the temporal chain of causality, we directly used the occupational earnings at job entry. The rapid economic development over the past 40 years since China’s reform and opening up has resulted in substantial price increases. Therefore, we used the Consumer Price Index (CPI) for each city in each year to eliminate the effects of inflation, allowing occupational earnings to be comparable across each generation of new workers. Moreover, due to the right-skewness of the original income distribution, after eliminating the effects of inflation, we then took the logarithm of the earnings to bring the distribution of earnings, the dependent variable, closer to a normal distribution.

**Job satisfaction**. Job satisfaction is a global indicator. We focused on job seekers at job entry and on the following five dimensions of satisfaction: the work itself, pay and benefits, opportunities for promotion, colleague relationships, and upper management. Each item was measured with a 5-point Likert scale ranging from “very unsatisfied” to “very satisfied”. We summed up the scores across the five items and found the average, which represents the overall satisfaction of job seekers with the results of their job search.

#### 3.2.2. Independent Variables

**The Use of Social Networks.** The use of social networks is both the most dominant independent variable in the model for predicting income (the regression model) and is the dependent variable in the model for predicting the likelihood of using social networks (the selection model). In coding the variables, “the use of social networks” was defined not only for those respondents who directly answered that they had used social networks when searching for their job but also for those who mentioned using social networks in other follow-up questions. This is because in Chinese social life, the use of social networks, i.e., the use of connections, is often seen as synonymous with “going through the back door”, which often has a negative connotation of unfairness. Some respondents were reluctant or hesitant to answer directly that they had used their social networks, but in the follow-up questions, they expressed indirectly that they had done so.

#### 3.2.3. Control Variables

We measured respondents’ attitudes toward relationalism by their willingness to “handle things through social networks”. This is a continuous variable, with a higher score indicating that respondents were more receptive to the idea of handling things through social networks and thus that they are more accepting of relationalism.

The control variables in this paper also include personal attributes, family background, and structural variables such as gender, age, *hukou*, education, Party membership, father’s education, father’s Party membership, labor market sector, an indicator for high-income industries, occupational requirements, and year of job entry. All variables used in this study can be found in Table 1.

### 3.3. Methods

To test whether the income return to social networks varies according to the propensity of the actors to use social networks when searching for a job, we used the stratification-multilevel method (SM model) [33] to develop our model for empirical analysis. Our approach is similar to propensity score subclassification [27] in that the whole sample was divided into a number of balanced subsamples, which were then analyzed in the next step. The specific analysis in this study followed four steps.

***Step 1*.** First, we needed to discuss the probability with which social networks are used during job search; this probability is unknown and needs to be estimated. We developed a logit regression model with a dummy variable (i.e., “whether social networks are used”) as the dependent variable, and the resulting predicted probability is the probability that the actors use their social networks, with higher values indicating a higher probability.

***Step 2*.** We constructed balanced propensity score strata following a principle that we call “homogeneity within groups, heterogeneity between groups”. Specifically, there should be no significant differences in the average values of covariates and in propensity scores between the treatment and control groups. In this study, “homogeneity within groups” means that the two types of job seekers, those who use social networks and those who do not, were randomly distributed across the subsamples defined by the propensity scores and that there were no significant differences in the distribution of those independent variables that influence the use of social networks. “Heterogeneity between groups” means that the different subsamples had significantly different probabilities of using social networks: the higher the stratification of a subsample, the more likely it is to represent the individuals in that subsample who use social networks.

***Step 3*.** Next, we estimated the propensity score stratum-specific treatment effects in terms of the instrumental (earnings) returns and emotional (job satisfaction) returns to social networks. We can eliminate the heterogeneity bias in the use of social networks because of our within-group homogeneity. We can directly use multiple linear regression models to estimate the earnings and emotional returns to social networks in each subsample, which means that we can obtain the treatment effects for each stratum.

***Step 4.*** Finally, we observed the relationship between the social network effects and the ranking of each subsample in terms of propensity scores. If the social network effects are greater in the higher ranked subsamples, then there is a positive selection effect related to the use of social networks in the labor market: the more likely someone is to use social networks, the higher the return to their social network is in terms of earnings or job satisfaction. In contrast, if the social network effects are lower in the higher ranked subsamples, then there is a negative selection effect, and the more likely people are to use social networks, the less rewarding the use of those social networks will be. Specifically, to conduct this analysis, we fit the data using a variance weighted least squares (VWLS) regression with the ranking of the subsamples as the independent variable and the social network effects for each subsample as the dependent variable. Since the sample for this step is simply the number of subsamples, the sample size was too small for direct estimation via ordinary least squares (OLS). VWLS uses standard deviations as weights, which relaxes the requirement for homoskedasticity and overcomes the small-sample problem of OLS.

## 4. Results

### 4.1. Descriptive Statistics

Table 1 presents descriptive statistics for the sample and compares both of the groups we focused on: those who use social networks when searching for a job and those who do not use social networks. The continuous variables (e.g., “log earnings”) are described in terms of their means, the categorical variables (e.g., “education”) are described in terms of percentages for all categories other than the reference category, and the last column shows the results of a significance test. Clearly, there were a number of significant differences between job seekers who use social networks and those who do not. Most notably, job seekers who use social networks had higher entry-level earnings than those who do not, with the former earning an average of 340 per month (exp (5.83)) and the latter earning only 226 (exp (5.42)). The same trend was observed for job satisfaction: job seekers who use social networks had a mean job satisfaction value of 3.6, while job seekers who did not use social networks when seeking employment had lower job satisfaction, a value of 3.47 (*p* = 0.036). However, it is not yet clear as to whether the differences in earnings and job satisfaction observed at this point are the result of job seekers’ use of social networks because the binary analysis indicates that the use of social networks when seeking a job is not a random event but is influenced by numerous demographic and social factors. For example, the average age of job seekers who use social networks was found to be 29 years old, which is significantly higher than that of job seekers who do not use social networks (26 years old); those who seek jobs in the private labor market sector were found to also more likely to use social networks than those in the public labor market sector. In addition, factors such as the year of job entry, household registration at the time of the job search, education, and attitudes toward relationalism, as well as occupational requirements, all affect whether social networks are used in the job search process.

### 4.2. Selectivity in Using Social Networks: The Logit Model

As the descriptive analysis in Table 1 shows, the distributions of the two types of job seekers, those who use social networks and those who do not, were not consistent across many variables. To obtain an unbiased estimate of the social network effect, we first looked at the selection in social network use: who is more likely to use their social networks in their job search? We constructed a logit model with the use of social networks in job search as the dependent variable in order to predict the likelihood of using a social network. The variables included in the model to control for factors influencing the use of social networks in job search were gender, age, household registration, education, Party membership, parental education, parental Party membership, work-unit sector, an indicator for high-income industries, occupational requirements, and the year of job entry. The results are shown in Table 2.

From the results shown in Table 2, it is easy to see that the use of social networks when seeking a job is indeed influenced by many factors, both personal characteristics such as age, education, household status, and attitudes toward relationalism, as well as structural factors such as labor market sector, occupational requirements, and the year of job entry. Again, this finding verifies that the use of social networks in job searching is not random but that those who use social networks share certain common behavioral characteristics [37]. People do not randomly become social network users or nonusers; rather, people with certain common characteristics become social network users. The logit model can provide a predicted probability of using social networks, and this probability value is important, as it is the basis for the stratification conducted in the next step.

### 4.3. Propensity Score Strata

On the basis of the predicted results of the logit model presented in Table 2, we divided the ranked predicted probability values (propensity scores) to generate six subsamples that were heterogeneous between groups but homogeneous within groups. These subsamples are referred to as strata one through six in Table 3. We divided the sample in descending order so that a higher stratum number indicates a higher likelihood of using social networks in the job search. Specifically, the probability that job seekers in the first stratum used their social networks in the job search process was the smallest—less than 0.1—and the number and proportion of job seekers who actually used social networks in this subsample was also the smallest, with only 84 out of 393 people using social networks. The probability of using a social network was slightly higher in the second stratum than in the first and ranged from 0.1 to 0.19. The number and proportion of job seekers who actually used social networks were also higher than those in the first stratum, with 124 out of 354 respondents using social networks. Finally, respondents in the sixth stratum had the highest probability of using a social network, with each person having a probability of more than 85%. The number and percentage of those who actually used their social networks were also the highest at 437 out of 589.

Each column in Table 3 represents a stratum, and each row represents an independent variable that helps predict the likelihood of using a social network. Ideally, within each stratum of the subsample, the effects of each independent variable on the dependent variable (the use of social networks) would be statistically insignificant, which means that the use of social networks is not confounded by any other factor in the subsample and can be considered a purely exogenous variable. However, in practice, it is very difficult to obtain such a perfect match, and there are many cases where the individual variables are still significant. For example, the variable “paternal Party membership” in the fifth stratum has a *p*-value of 0.044, indicating that in this stratum, paternal Party membership still influences whether respondents use their social networks. As suggested by Xie and his colleagues [33,34], we need to further control for these still significant variables in the next step of the model when we predict the effect of social networks.

After obtaining the six propensity score strata, we then calculated the effects of the use of social networks in each stratum and subsequently fit the relationship between the number of strata (the propensity to use one’s network) and the effects of social networks using VWLS. The difference between VWLS and OLS is that VWLS relaxed the assumption of homoskedasticity. In fact, the variances did vary across the different subsamples. In addition, we also found the average treatment effect for the overall sample, following the conventional method for propensity score matching. The final results of the analysis are presented in Table 4 (analysis of heterogeneous effects of social networks on earnings) and Table 5 (analysis of heterogeneous effects of social networks on job satisfaction).

Turning first to Table 4, the table is divided into three panels. The top panel reports the coefficient, standard error, and *Z* value of the social network effect for each of the six subsamples. As the subsample ranking increased (i.e., as the stratum number increased), the effect of social networks on earnings became progressively smaller and eventually become negative (from 0.306 to −0.054). It should be mentioned that because the subsamples in each stratum were created according to the empirical stratification results, the subsamples themselves were not representative, and the results for those subsamples were not generalizable to higher-level groups. In fact, this step is only an intermediate step in the entire analysis process, and the results are only meaningful after weighted summation as in propensity score stratified matching or regression fitting using variance weighting as in the SM model.

The middle and bottom panels of Table 4 present the results of a composite analysis based on the effects of each stratum. The middle panel presents the calculated average earnings effect of social network use for the overall sample according to the propensity score subclassification (PSS) method.

b=∑k=1K[(nkN)∗bk]Var(b)=∑k=1K[((nkN)^2)∗Var(bk)]SE(b)=Var(b)Z=b/SE(b)

where *b* is the average effect of social network use, k = 1, 2, …, 6 indexes the number of strata, $b_{k}$ is the coefficient representing the effect of social network use on earnings in each stratum, $n_{k}$ is the number of observations in each stratum, N is the number of observations in the whole sample, and *SE*(*b*) is the standard error of *b*. When the average treatment effect *b* is divided by its standard error, *SE*(*b*), the *Z* value used for statistical inference is obtained. In the overall sample, job seekers who use social networks had significantly higher earnings than those who do not (coefficient of 0.031, *Z* > 1.96), which is consistent with the findings of previous studies.

The bottom panel of Table 4 shows the relationship between the social network earnings effect and the propensity to use social networks estimated via VWLS. The results indicate that there was a significantly negative relationship (−0.045) between the strata ranking and the social network effect.

Table 5 is structured in the same way as Table 4. Looking at the top of the table reveals that within each subsample, the coefficient representing the effect of social network use on job satisfaction showed steadily increases. The results in the middle panel were calculated in the same way as the earnings effect and indicate that in the overall sample, there was no significant difference in job satisfaction between job seekers who use social networks and those who do not. The bottom panel of the table shows the relationship between the effect of social networks on job satisfaction and the propensity to use social networks obtained via VWLS. The results show that there was a significant positive effect (0.030) between the strata ranking and the social network effect, and this effect was statistically significant. That is, the higher the propensity to use social networks when seeking employment, the higher the job satisfaction of job seekers. This result is the opposite of that for the earnings effect.

## 5. Conclusions and Discussion

The job search process for laborers is actually the process of allocating labor resources through the labor market. From the planned economy in China’s pre-reform period to its dual-track system at the beginning of the reform and then into the era of the market economy as the reform deepened, resources have been allocated through three channels: redistribution, the market, and social networks [4,17,19,38]. Of these, redistribution channel and the market channel are formal channels for resource allocation, while the social network channel is an informal resource allocation channel. Theoretically, as marketization increases over time, market rules become increasingly clear, institutional rigidity becomes stronger, the power of formal channels increases, and the use of informal channels such as social networks should decrease. However, the most recent empirical data show that the proportion of workers using social networks during the job search process has increased to nearly 80% of the samples [39]. These realistic data suggest that the use of social networks in job searching is no longer restricted to a special group, and the internal heterogeneity within the group of social network users is gradually increasing [40]. This leads us to ask: for this internally heterogeneous group, is there also variability in the effect of social networks? That is, are the returns to social networks consistent across those job seekers who use them? Focusing on the use of and returns to social networks during job searching, this paper attempts to address the two-part endogeneity issue: First, the endogeneity in pretreatment effects, which means whether the use of social networks is a random event; second, the endogeneity in treatment effects, in this study meaning whether the return to social networks is consistent among those actors who use them when searching for a job. We employed a large set of survey data on social networks and used a heterogenous treatment effects model in our empirical analysis. We obtained the following main conclusions:

First, the use of social networks in job searching is not random, and the propensity of job seekers with different characteristics to use social networks is not the same. In the process of job searching, which is an instrumental action, the choice of job seekers to use or not to use social networks is not a random event. Rather, there are certain characteristics that lead some people to use their social networks, while others choose not to. These characteristics are both individual and structural.

Most previous research on social network effects has been based on analyses of regression models. Research based on OLS assumes that all the behaviors of the study occur randomly; however, the creation and the use of social networks—interactions among actors—is purposeful and highly subjective. It is difficult to satisfy the requirement of random distribution [22,23]. In our empirical analysis, we found that there do indeed exist some shared characteristics that result in essential differences between the two types of people: those who use social networks and those who do not.

Second, the returns to social networks are different for job seekers with different propensities to use social networks. This means that in the labor market job search process, there are two different selection biases working at the same time: there is not only pretreatment heterogeneity, which means that the use of social networks suffers from selection bias, but also treatment effect heterogeneity, which means that the effects of social networks are also biased due to endogeneity. Even within the group of those who use their social networks when searching for a job, the returns to that use differ from person to person.

Third, there is negative selection in the instrumental effect of social network use in the job search process but positive selection in the expressive effect: the higher the propensity to use one’s networks, the lower the income return to social networks and the higher the job satisfaction; the lower the propensity to use one’s networks, the higher the income return and the lower the job satisfaction.

Endogeneity has been a major challenge in social network analysis in recent years, especially in research on social network effects in labor markets [22,23,41]. Scholars have tried to respond to this endogeneity challenge, which takes many forms, such as the homogeneity of social networks [24], the selectivity of social networks [12,13,37], and reverse causality [42]. Previous studies on social network effects have not found heterogeneous returns to the use of these networks, which may have been due to the limitations of their research methodology. This study identified endogeneity in both pretreatment effects and treatment effects and estimated the effect of social network use while considering the complete endogeneity problem. People differ not only in their use of social networks but also in how they are affected by that use. This study provides ideas for analyzing the heterogenous returns to social networks. We first estimated the probability of using social networks and then stratified the sample on this basis to calculate the effects of social networks among those with different propensities to use their social networks. Finally, we tested the relationship between the ranking of the strata and the effect of social network use.

Nan Lin has reviewed 31 studies on the relationship between social capital and status attainment from the last two decades of the last century, and 30 of those studies demonstrated a link between social capital and status attainment [17]. Are these links the same for different actors? Nan Lin’s strength-of-position proposition states that the better the initial position of an actor is, the more likely they are to access and mobilize higher levels of social capital. This study builds on this proposition and further explores the differences in returns related to different propensities. The results show that in a pyramidal social structure, there are not only differences in the distribution of resources but also differences in the returns to resources.

## 6. Limitation

This paper also has some limitations. In this study, we first explored the heterogeneity in the returns to social networks; however, more advanced research may be conducted in the future. In our exploration of the use of social networks, we used various external individual-level characteristics to comprehensively analyze the propensity to use social networks. We believe that a third dimension could be introduced in future research that enables an in-depth exploration of the relationship between group characteristics and the propensity to use social networks and the differences in the corresponding returns. For example, information about class [43], a concern of sociological research, could be included. Are there differences in the propensity of members of different classes to use their social networks, and are there differential returns? This would further enrich the social implications of social network research.

Second, this study was concerned with the returns to social networks at the beginning of the job seeker’s entry into their career. In addition to the role of social networks in the job search process, which is to help job seekers find jobs and earn high salaries, social networks may also have long-term effects and delayed returns. In the real labor market, job seekers’ use of social networks to obtain jobs may not necessarily generate benefits at the beginning of their entry into the workforce. Whether those who find jobs through social networks are sheltered by that network after they enter their job, thus obtaining long-term professional development, rapid promotions, and job security, is an interesting question [44]. The collection of panel data will help us answer this question effectively.

In addition, this study was concerned with the use of and returns to social networks in the labor market, and the labor market segmentation model is a classical theoretical model in labor market research. The object of analysis in this paper was the use of social networks in the labor market as a whole. A prominent feature of China’s social development in the past 40 years since its reform and opening up is inequality. What kind of differences might exist in the use of and returns to social networks in different periods and regions? Whether institutional differences, industrial differences, and differences in occupational levels have caused labor market segmentation for different reasons and whether this segmentation has led to differences in the use of and returns to social networks are specific topics to be discussed in depth in future research.

## Figures and Tables

**Table 1:** Variable description table.

	All Samples	Not Use SN	Use SN	*p*-Value
Earning (logged)	5.63	5.42	5.83	0.000
Job satisfaction	3.63	3.47	3.60	0.036
Age	27.6	26	29	0.000
Gender (ref: female)	0.458	0.464	0.452	0.457
Era of job entry (ref: 1978–1992)				0.000
1993–2002	0.182	0.182	0.182	
2003–2014	0.582	0.478	0.675	
Household registration (ref: rural)	0.835	0.875	0.799	0.000
Education (ref: below middle school)				0.001
High school	0.273	0.274	0.272	
College (no degree)	0.245	0.255	0.236	
Tertiary education	0.307	0.322	0.294	
Party membership	0.104	0.108	0.101	0.481
Parental education (ref: below primary school)				0.138
Middle school	0.219	0.216	0.221	
High school	0.275	0.272	0.278	
Tertiary education	0.117	0.108	0.125	
Not clear	0.048	0.056	0.042	
Parental Party membership	0.238	0.238	0.238	0.980
Labor market sector (ref: private)	0.524	0.609	0.447	0.000
Attitudes of relationalism	3.27	3.23	3.31	0.000
Occupational requirements	0.55	0.526	0.575	0.002
No. of cases	3930	1857	2073	

**Table 2:** Logit model predicting use of social networks or not.

	Coefficient	S.E.
Age	0.024 ***	(0.005)
Gender (ref: female)	−0.047	(0.068)
Era of job entry (ref: 1978–2001)		
1993–2002	0.785 ***	(0.114)
2003–2014	1.061 ***	(0.110)
Household registration (ref: rural)	−0.389 ***	(0.099)
Education (ref: below middle school)		
High school	−0.249 **	(0.110)
College (no degree)	−0.603 ***	(0.123)
Tertiary education	−0.708 ***	(0.128)
Party membership	−0.093	(0.114)
Parental education (ref: below primary school)		
Middle school	0.185	(0.168)
High school	0.208	(0.176)
Tertiary education	0.293 *	(0.177)
Not clear	0.557 ***	(0.195)
Parental Party membership	0.134	(0.084)
Labor market sector (ref: private)	−0.209 ***	(0.075)
Attitudes of relationalism	0.244 ***	(0.057)
Occupational requirements	0.165 **	(0.074)
Constant	−1.571 ***	(0.292)
No. of cases	3931	

S.E. for Stand Error; * *p* < 0.05; ** *p* < 0.01; *** *p* < 0.001.

**Table 3:** Propensity score strata of using social networks.

	Stratum 1	Stratum 2	Stratum 3	Stratum 4	Stratum 5	Stratum 6
	Min–0.1	0.1–0.19	0.19–0.42	0.42–0.65	0.65–0.85	0.85–Max
Age	0.587	0.724	0.434	0.178	0.804	0.524
Gender	0.292	0.095	0.676	0.269	0.310	0.983
Era job entry	0.939	0.735	0.126	0.234	0.560	0.148
Education	0.639	0.241	0.281	0.837	0.916	0.042
Party membership	0.765	0.763	0.310	0.512	0.079	0.669
Household registration	0.939	0.794	0.790	0.303	0.311	0.416
Attitudes of relationalism	0.949	0.315	0.820	0.980	0.292	0.742
Parental education	0.123	0.163	0.346	0.923	0.333	0.990
Parental Party membership	0.618	0.242	0.573	0.625	0.044	0.199
Labor market sector	0.150	0.241	0.067	0.293	0.601	0.416
Occupational requirements	0.532	0.596	0.358	0.114	0.921	0.593
No. of cases	393	354	904	904	786	589
Using SN = 1	84	124	420	488	520	437

**Table 4:** Heterogeneous effects of social network on earnings.

Network Effects within Strata				
Strata	No. of Cases	Coefficient	S.E.	*Z* Value
1	393	0.306	0.136	2.250
2	354	0.068	0.115	0.591
3	904	0.037	0.059	0.627
4	904	−0.040	0.054	−0.740
5	786	−0.017	0.062	−0.271
6	589	−0.054	0.066	−0.827
**Average Effect of Social Network in Overall Sample**				
(Propensity Score Subclassification)				
		0.031	0.014	2.25
**Heterogeneous Effects of Social Network on Earnings**				
(Variance—Weighted Least Squares)				
		−0.045 *	0.021	−2.11

* *p* < 0.05.

**Table 5:** Heterogeneous effects of social network on job satisfaction.

Network Effect within Strata				
Strata	No. of Cases	Coefficient	S.E.	*Z* Value
1	390	−0.149	0.076	−1.960
2	351	0.075	0.071	1.054
3	903	−0.027	0.040	−2.686
4	904	−0.036	0.038	−0.935
5	782	−0.003	0.044	−0.075
6	586	0.133	0.060	2.207
**Average Effect of Social Network in Overall Sample**				
(Propensity Score Subclassification)				
		−0.015	0.020	−0.722
**Heterogeneous Effects of Social Network on Job Satisfaction**				
(Variance—Weighted Least Squares)				
		0.030 *	0.015	1.98

* *p* < 0.05.

## Data Availability

The data presented in this study are available on reasonable request from the corresponding author.
